# It’s SNARC o’ clock: manipulating the salience of the context in a conceptual replication of Bächtold et al.’s (1998) clockface study

**DOI:** 10.1007/s00426-023-01893-x

**Published:** 2023-10-25

**Authors:** Serena Mingolo, Valter Prpic, Alberto Mariconda, Peter Brugger, Thekla Drack, Eleonora Bilotta, Tiziano Agostini, Mauro Murgia

**Affiliations:** 1 Department of Humanities, University of Trieste, Trieste, Italy; 2 Department of Philosophy and Communication Studies, University of Bologna, Bologna, Italy; 3 Institute for Psychological Science, De Montfort University, Leicester, UK; 4 Department of Life Sciences, University of Trieste, Trieste, Italy; 5 Neuropsychology Unit, University Hospital Zürich, Zurich, Switzerland; 6 Neuropsychology Unit, Rehabilitation Center Valens, Valens, Switzerland; 7 Department of Physics, University of Calabria, Rende, CS Italy

## Abstract

The Spatial-Numerical Association of Response Codes (SNARC) effect consists in faster left-/right-key responses to small/large numbers. (Bächtold et al., Neuropsychologia 36:731–735, 1998) reported the reversal of this effect after eliciting the context of a clockface—where small numbers are represented on the right and large numbers on the left. The present study investigates how the salience of a particular spatial-numerical context, which reflects the level of activation of the context in working memory, can alter Spatial Numerical Associations (SNAs). Four experiments presented the clockface as context and gradually increased its salience using different tasks. In the first two experiments (low salience), the context was presented at the beginning of the experiment and its retrieval was not required to perform the tasks (i.e., random number generation in Experiment 1, magnitude classification and parity judgement in Experiment 2). Results revealed regular left-to-right SNAs, unaffected by the context. In Experiment 3 (medium salience), participants performed magnitude classification and parity judgement (primary task), and a Go/No-go (secondary task) which required the retrieval of the context. Neither the SNARC effect nor a reversed-SNARC emerged, suggesting that performance was affected by the context. Finally, in Experiment 4 (high salience), the primary task required participants to classify numbers based on their position on the clockface. Results revealed a reversed SNARC, as in (Bächtold et al., Neuropsychologia 36:731–735, 1998). In conclusion, SNARC is disrupted when the context is retrieved in a secondary task, but its reversal is observed only when the context is relevant for the primary task.

## Introduction

Spatial-Numerical Associations (SNAs) are among the most important examples of the overlap between space and number representation in human cognition. Among SNAs, the SNARC effect (Spatial-Numerical Association of Response Codes; Dehaene et al., 1993) is paradigmatic and has most frequently been investigated (for a meta-analysis, see Wood et al., 2008). This effect consists in the facilitation, exhibited by people from Western cultures, to respond to a small number with a left key and to a large number with a right key. This facilitation in response execution applies both to speed and accuracy. Dehaene et al. (1993) suggested that the SNARC effect can be attributed to the long-term representation of magnitudes on a “Mental Number Line” (MNL; Restle, 1970), in which small numbers are associated to the left side of the line and large numbers are associated to the right. According to this account, the SNARC effect would originate from a long-term association between numbers and space.

Despite the MNL account is well-known in SNAs research, some studies seem to challenge it. In particular, growing evidence suggest that the relation between numbers and space can be constructed temporarily during task execution (Fias & van Dijck, 2016), which implies a crucial involvement of working memory. Proofs of the involvement of working memory were provided in a seminal study by van Dijck et al. (2009), who found that the SNARC effect depends on the working memory resources available at a given moment. In their experiments, the SNARC effect disappeared under a visuospatial working memory load in magnitude comparison and under a verbal working memory load in parity judgment. In another study, participants were asked to perform a parity judgement on a sequence of random numbers that they had previously memorized (van Dijck & Fias, 2011). Results showed an “ordinal position effect”, namely an association between the ordinal position of items in the memorized sequence and the response coordinates (i.e., first items of the sequence were associated to the left and last items to the right, regardless of numbers’ magnitude). According to these studies, the SNARC effect cannot be explained by long-term, immutable associations between numbers and space, rather it seems that working memory plays a crucial role in regulating these associations depending on task requirements.

The SNARC effect and, more in general, SNAs can be observed in a variety of different tasks. Most common are magnitude classification and parity judgement tasks. In magnitude classification, participants are required to classify a centrally presented number (e.g., 2) as smaller or larger than a fixed reference (e.g., 5) by pressing either a left or right key, depending on the condition. In parity judgement, participants are required to classify a centrally presented number (e.g., 3) as even or odd, by pressing either a left or right key, depending on the condition. Magnitude classification is considered a “direct task”, because it requires participants to directly compare a feature of the stimuli relevant for the study (i.e., magnitude) with a reference. Conversely, parity judgement is considered an “indirect task”, because participants are asked to judge a feature of the stimuli irrelevant to the study, namely parity (Mingolo et al., 2021).

Another task that has been used to investigate spatial biases in number processing is the random number generation task (RNG). This task requires participants to continuously enumerate numbers included in a given numerical interval, usually in combination with spatial instructions. This task revealed that people generally produce more small numbers when turning their head to the left, and more large numbers when turning their head to the right (Loetscher et al., 2008). Similarly, higher production of small numbers was found after spontaneous downward/leftward eye movements, together with higher production of large numbers after upward/rightward eye movements (Loetscher et al., 2010). More in general, a tendency to generate significantly more small numbers than the chance level has been observed in healthy subjects (Loetscher & Brugger, 2007). This preference is referred to as “small number bias” (SNB), and it has been attributed to a leftward bias defined as “pseudoneglect”. This bias would lead healthy subjects to preferably allocate their attention to the left side of the MNL when processing numbers (Loetscher & Brugger, 2009).

The SNARC effect has been observed not only using different tasks, but also using different kinds of stimuli. Indeed, this effect does not limit to numerals. Other stimuli conveying a quantity exhibited SNARC-like effects, such as objects’ size (Prpic et al., 2020; Ren et al., 2011; Sellaro et al., 2015), luminance (Fumarola et al., 2014; Ren et al., 2011), and angle magnitude (Fumarola et al., 2016). Similarly, ordinal stimuli such as weekdays, months, letters (Gevers et al., 2003, 2004) and musical notation (Fumarola et al., 2020) are spatially mapped. The SNARC effect is very consistent and has been replicated not only with different kinds of stimuli, but also with different presentation modalities like the auditory (Bruzzi et al., 2017; De Tommaso & Prpic, 2020; Hartmann & Mast, 2017; Lega et al., 2020; Mariconda et al., 2022; Prpic & Domijan, 2018) and somatosensory ones (Dalmaso & Vicovaro, 2019; Vicovaro & Dalmaso, 2021).

Although the SNARC effect is robust and replicable, a large amount of evidence indicates that this effect is quite flexible. The effect can be influenced by different experiences such as the reading/writing direction of participants (Cipora et al., 2019; Dehaene et al., 1993; Shaki et al., 2009; Zebian, 2005; Zohar-Shai et al., 2017), by activities that spatialize numbers in our daily lives, such as finger counting (Fischer, 2008; Hohol et al., 2021; Pitt & Casasanto, 2020), and by the context in which numerical stimuli are presented (Bächtold et al., 1998; Mingolo et al., 2021).

Famously, the study by Bächtold et al. (1998) showed that the context in which number are presented has the potential to alter, and even reverse, the SNARC effect. In that two-part study, participants were instructed to conceive centrally presented numbers (ranging from 1 to 11) as distances on a ruler (thus evocating the MNL), and to judge whether such distances were shorter or longer than 6 cm. Results were in line with the SNARC effect, with faster left-key responses to small numbers and faster right-key responses to large numbers. In a second experiment, other participants did the same task after being exposed to a clockface context, whose spatial representation of numbers is opposite to the MNL (i.e., small numbers are on the right and large numbers are on the left). In this experiment, faster right-hand responses to small numbers and faster left-hand responses to large ones were observed, showing that the clockface context led to a reversal of the traditional SNARC effect.

A recent study tested the influence of context on the SNARC effect using a mobile-phone keypad (Mingolo et al., 2021). The consistency between the representation elicited by the context and by the task was manipulated using three different tasks. The context shaped a keypad-like SNA when the task elicited a representation consistent with the one elicited by the context. However, an influence of the context emerged at a certain degree in other tasks as well. Overall, from the literature it is not clear whether these results are due to the context alone or to the salience of the context as determined by the task.

The present study aims to clarify how context may alter typical left-to-right SNAs. Furthermore, we investigate how task demands modulate the salience of the context (which reflects the level of activation of the context in working memory), and thus its effect on SNAs. To achieve these goals, the effect of the context employed by Bächtold et al. will be systematically investigated in different tasks. The clockface context will be kept constant across the experiments, while task demands will be modified to gradually increase the level of salience of this context (low, medium, high).

In the first part of the study (Experiment 1 and Experiment 2), the clockface context is elicited at the beginning of the experiments, while task instructions are completely unrelated to it. In this way, task demands will induce a low level of salience of the context. The tasks used are a random number generation (RNG) task in Experiment 1, and two classical SNARC tasks in Experiment 2 (magnitude classification and parity judgement). In the second part of the study (Experiment 3), the clockface context is introduced at the beginning of the experiment and participants perform a dual task. In particular, the primary tasks consist, as in Experiment 2, in a magnitude classification and a parity judgement. The secondary task reinforces the salience of the context using a Go/No-go procedure. This procedure is meant to retrieve in working memory the contextual configuration processed at the beginning of the experiment. In this way, task demands will induce a medium level of salience of the context. Finally, in the last part of the study (Experiment 4), the clockface context is introduced at the beginning of the experiment, and the instructions of the primary task are directly based on it. The task requires participants to classify numbers depending on their spatial position on the clockface. In this way, task demands will require the retrieval of the contextual configuration from working memory, thus inducing a high level of salience of the context.

## Experiment 1

Experiment 1 investigates the effect of the clockface context—introduced at the beginning of the experiment—on the small-number bias (SNB). SNB indicates the tendency to produce more small numbers than large numbers (in a given numerical interval) during RNG tasks. This effect would be explained by a ‘pseudoneglect in number space’ exhibited by healthy participants (Loetscher & Brugger, 2007). Pseudoneglect is the tendency to preferentially attend to the left side of space. It can be found, for instance, in traditional bisection tasks, where participants tend to misplace the midpoint to the left of its exact position (Jewell & McCourt, 2000). It has also been demonstrated in “number line bisections”, where participants (of Western cultures) tend to misplace the numerical midpoint of two given numbers towards smaller numbers, i.e., to the left on the MNL (e.g., Brugger et al., 2010).

The aim of Experiment 1 is to investigate whether RNG reveals the presence of pseudoneglect when the same configurations employed by Bächtold et al. (1998) are used as context: the clockface configuration (Experiment 1a) and the ruler configuration (Experiment 1b). If this is the case, numbers placed left in context-dependent representational space should be overrepresented in both configurations. This would mean a reversal of the SNB (i.e., an overrepresentation of large numbers) in the clockface configuration, and the typical SNB in the ruler configuration.

### Method

#### Participants

We tested 35 participants (20 women, 15 men) with a mean age of 30.53 (SD = 10.27). The sample size was determined by means of the software MorePower 6.4. The following parameters were used: power = 0.80, α = 0.05, Cohen’s d = 0.44 (the effect size was extracted from Winter & Matlock, 2013) ; the outcome was a suggested sample size of 34 participants. All participants reported to be right-handed, to have normal or corrected-to-normal vision and to have always been used to exclusively read and write in a left-to-right direction. Before the experiment, participants provided written informed consent to participate to the study. The present experiment was conducted in accordance with the ethical standards indicated by the Declaration of Helsinki and with the approval of the University of Trieste Ethics Committee.

#### Apparatus and stimuli

**Fig. 1:**
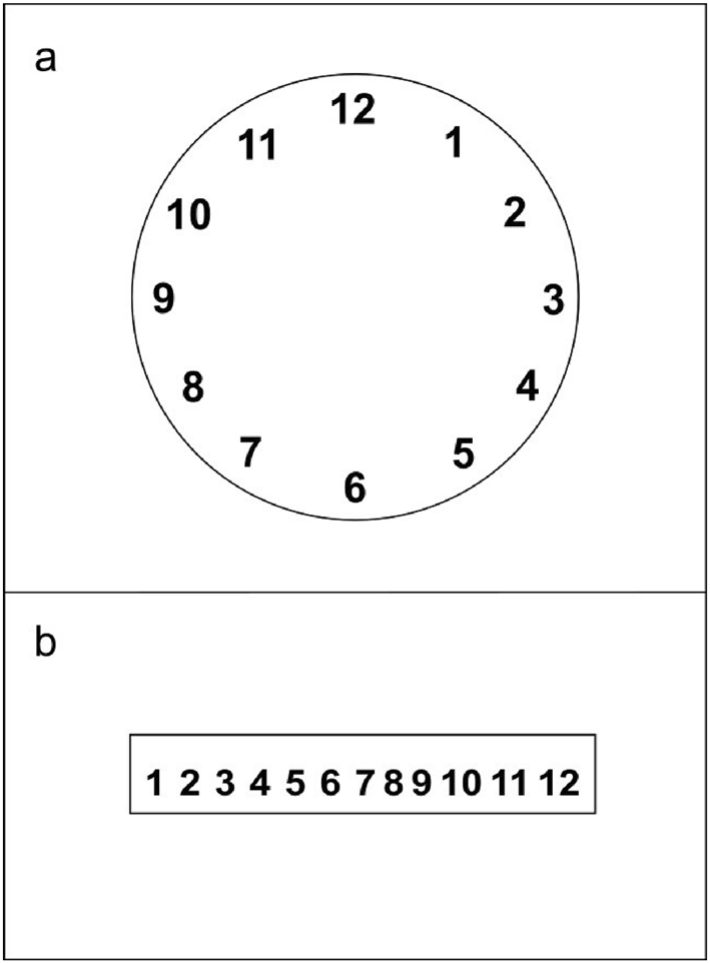
The clockface (**a**) and the ruler (**b**) presented at the beginning of Experiments 1a and 1b, respectively

A Dell desk computer with Intel Core i5 (RAM: 4 Gb) was employed to prepare two images, one representing a clockface (Fig. 1a) and the other representing a ruler (Fig. 1b). They were displayed on a Quato Intelli Proof 242 excellence (24 inches) monitor, with a 1024 × 768 resolution. A metronome and a tape recorder were used.

#### Procedure

In Experiment 1a, participants sat on a chair located in front of the screen, with their body aligned to the screen’s midline. When the participant was ready, the clockface picture (Fig. 1a) was presented at the centre of the screen for 20 s and participants were asked to pay particular attention to it. After presentation of the clockface picture, participants were asked to close their eyes and to imagine the picture while performing RNG. This task required to vocally produce 60 numbers in the range of 1–12 at the constant rhythm of 0.5 Hz, paced with the beat of a metronome. Numbers had to be generated in a sequence as random as possible, taking into consideration that any number could be followed by any other number with a comparable probability in the long run. Participants’ responses were tape-recorded during the task, to allow later annotation of the generated numbers. Experiment 1b followed the exact same procedure as Experiment 1a, but the ruler picture was presented instead (Fig. 1b). Every participant performed both Experiment 1a and 1b; the order of execution of the experiments was counterbalanced among participants.

### Data analyses and results

The number of times each number was generated was counted for each participant in each experiment. The numbers generated were labelled “small” (i.e., 1–2-3–4–5) or “large” (i.e., 7–8-9–10–11).

**Fig. 2:**
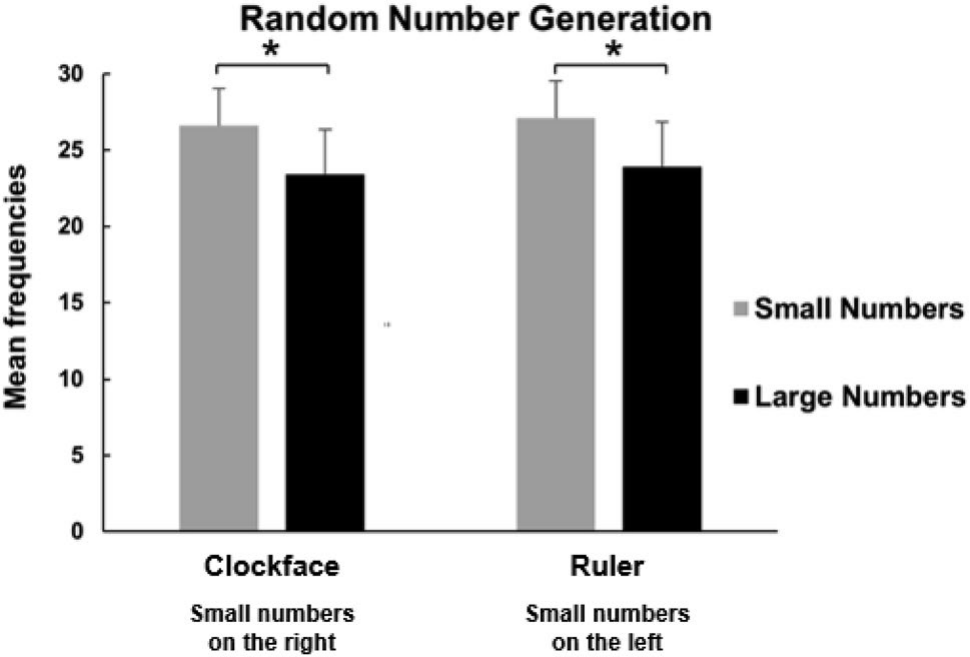
Mean frequencies of small vs. large numbers generated in Experiment 1a (clockface) and Experiment 1b (ruler). A significant difference was found in both configurations. Errors bars indicate the standard error of the mean

In Experiment 1a (clockface), small (rightward) numbers were generated more often than large (leftward) numbers (small numbers: M = 26.60, SD = 2.43; large numbers: M = 23.40, SD = 2.67; Fig. 2). A paired-sample *t* test showed that this difference was significant [*t*(34) = 4.05; *p* < 0.001; *d* = 0.68].

In Experiment 1b (ruler), small (leftward) numbers were generated more often than large (rightward) numbers (small numbers: M = 27.1, SD = 2.94; large numbers: M = 23.9 times, SD = 2.87; Fig. 2). A paired-sample *t* test showed that this difference was significant [*t*(34) = 3.51; *p* < 0.005; *d* = 0.59].

### Discussion

Results from Experiment 1 clearly indicate the presence of a SNB in both configurations. This evidence can be interpreted in two different ways: either the SNB is not determined by pseudoneglect, or the clockface context presented at the beginning of the experiment was not strong enough to produce an overrepresentation of large numbers.

The latter explanation is partially consistent with the results obtained by Mingolo et al. (2021) with the keypad context. Indeed, Mingolo et al. showed that context does not reverse the direction of SNAs as long as it is exclusively introduced at the beginning of the experiment. In this case, the lack of effect of the context on SNB could be because the task did not require to retrieve the context in working memory. This did not lead to the creation of a new SNA consistent with the clockface context since there was no strategical advantage in doing so.

The study by Mingolo et al. (2021) however shows that the context can affect SNAs to a certain degree, depending on particular task demands. To further explore how the clockface context affects SNAs we decided to run further experiments with the same tasks employed in their study, using the clockface instead of the keypad configuration.

## Experiment 2

Experiment 2 investigates the effect of the clockface context—introduced at the beginning of the experiment—on the SNARC effect. Some studies reported alterations of the SNARC effect due to a manipulation performed at the beginning of the experiment, although results are not always consistent. For instance, Fischer et al. (2010) manipulated the position of numbers in the context of written recipes. When numbers were located in a position that was incongruent with the SNARC effect (i.e., small/large numbers on the right/left side of the page), a reduction of the SNARC effect was observed, thus revealing an influence of the context on SNA. Similarly, Shaki and Fischer (2008) asked Russian/Hebrew bilinguals to read a text in Cyrillic (left-to-right) or Hebrew (right-to-left) before performing a parity judgment. They observed a regular SNARC after activating the left-to-right reading direction and a reduction of SNARC after activating the right-to-left reading direction. Furthermore, Mingolo et al. (2021) reported that the context prevented the SNARC effect in magnitude classification, but not in parity judgment.

In Experiment 2, the effect of the clockface context is tested in two typical SNARC tasks: magnitude classification (Experiment 2a) and parity judgement (Experiment 2b). In absence of trainings or context manipulations, these tasks typically reveal a regular SNARC effect. If the context elicited at the beginning of the experiment is salient enough, it should affect this expected pattern.

### Method

#### Participants

We tested 35 participants (28 women, 7 men) from the University of Trieste with a mean age of 19.80 (SD = 1.95). The sample size was determined by means of the software MorePower 6.4. The following parameters were used: power = 0.80, α = 0.05, Cohen’s d = 0.45 (estimated effect size from the average of the three most relevant experiments: Mingolo et al., 2021, Exp. 2a and Exp. 2b, and Bächtold et al., 1998); the outcome was a suggested sample size of 32 participants.

On being questioned, all participants reported to be right-handed, to have normal or corrected-to-normal vision and have always been used to exclusively read and write in a left-to-right direction. All participants reported that their psychophysiological state was not affected by alcohol consumption or insufficient sleep in the last 24 h (Murgia et al., 2020). Written informed consent was obtained by all participants. The present experiment was conducted in accordance with the ethical standards indicated by the Declaration of Helsinki and with the approval of the University of Trieste Ethics Committee.

#### Apparatus and stimuli

The experiment was designed and run through the Psychopy software, version 3.0, on the same computer and monitor employed in Experiment 1. A five-button serial response box was used to collect participants’ responses.

Stimuli consisted of ten numbers, i.e., 1–2-3–4–5–7–8–9-10–11, and were presented in the centre of the screen, one at a time and in randomized order, in white against a grey background. Stimulus numbers thus consisted of the numbers displayed on a standard clockface. Numbers 6 and 12 were not included as both take a position on the vertical midline through the clockface and are not associated with either left or right half of a clockface.

#### Procedure

The experiment took place in a quiet, dimly illuminated room. Participants were asked to sit comfortably and to move as little as possible, aligned to the midline of the PC screen, at a viewing distance of approximately 60 cm from it. They were instructed to put their left index finger on the leftmost key of the response box in front of them and their right index on the rightmost key.

Each participant performed two different tasks: magnitude classification in Experiment 2a and parity judgement in Experiment 2b. The order of presentation of the two tasks was counterbalanced among participants. Each task was split into two blocks (block A and block B), each including a practice session consisting of 50 trials (not considered for data analysis) and an experimental session (150 trials).

**Fig. 3:**
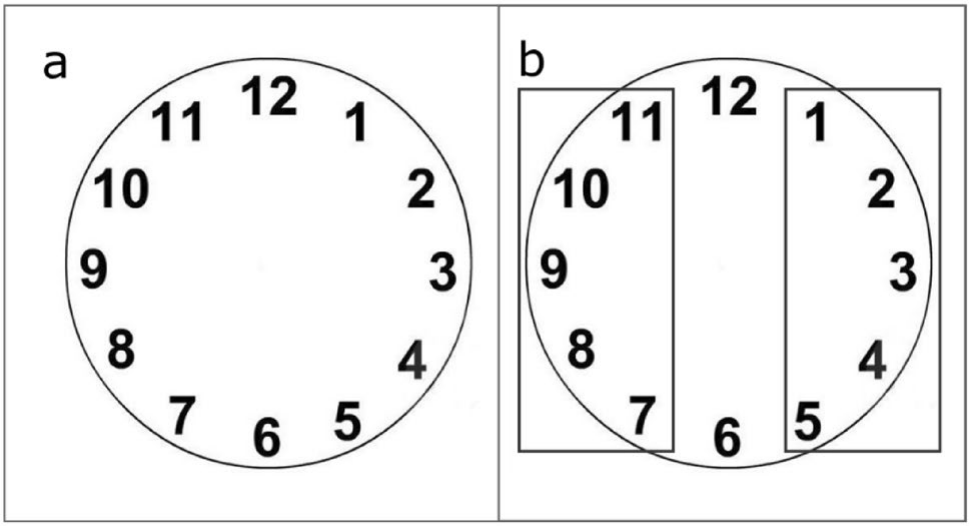
The clockface as presented to participants before the beginning of Experiment 2. Clockface exposure lasted 20 s. During the first 10 s only the clockface as shown in (**a**) was exposed and participants were instructed to watch it and to pay particular attention to it. During the following 10 s the two rectangles in (**b**) were superimposed, to highlight the numbers placed on the left and on the right side of the clockface

Before the beginning of each block, participants were exposed for 20 s to the picture of a clockface (Fig. 3a) and were instructed to look at the display and to pay particular attention to the spatial arrangement of the numbers. In the last 10 s of presentation of the clockface, two rectangles appeared on the left and right portion of the clockface, to highlight the numbers in those positions (Fig. 3b). Participants were instructed to keep an image of the clockface in mind during the entire experiment.

The practice session was divided into two parts. The first part (20 trials) started with a fixation cross (500 ms) followed, after an interstimulus interval (ISI) of 500 ms, by the picture of the clockface at fixation for 2000 ms. When the clockface picture disappeared, a fixation cross was presented for 500 ms, followed by an ISI of 500 ms. Finally, the target stimulus (a single-digit number) appeared in place of the fixation cross, until a response occurred (within a response time deadline of 2000 ms). Participants responded by pressing the leftmost or the rightmost key of the response box. The combination of the response buttons was reversed from block A to block B, and the order of presentation of the two blocks was counterbalanced among participants.

In the magnitude classification task (Experiment 2a), participants had to judge whether the presented number was smaller or larger than 6. In the parity judgement task (Experiment 2b), participants had to judge whether the presented number was even or odd. In this phase of the practice session, feedback about the response was given at each trial (“Correct!” or “Wrong!”). The second part of the practice session (30 trials) followed the same procedure as the first one but did not present the clockface at the beginning of the trial.

The experimental session (150 trials) followed the same procedure as the second part of the practice session, but without any feedback. Participants could decide to take a short break between the two blocks or to continue with the experiment. Instructions explicitly asked participants to be as accurate and as fast as possible.

### Data analysis and results

The independent variables were Hand (left vs. right) and Number (1–2-3–4–5–7–8–9–10–11), the dependent variable was the Response Time (RT). RTs of incorrect trials were not included in data analysis. Similarly, RTs shorter than 150 ms or those that differed by more than 2.5 standard deviations from a participant’s mean RT were considered outliers and removed from data analysis. In Experiment 2b, five participants were excluded because less than half of their RTs in at least one condition could be considered for the analyses. Then, mean RTs of the correct trials for the left and for the right hand were computed separately for each participant in each experimental session. Finally, to obtain the dRTs, the mean RTs of the left hand were subtracted to the mean RTs of the right hand: dRT = RT (right hand)—RT (left hand). Hence, positive dRTs indicate faster responses with the left hand, whereas negative dRTs indicate faster responses with the right hand.

A repeated measures ANOVA was performed on RTs for both experiments. To determine if the SNARC effect emerged, a regression analysis was conducted (Fias, 1996; Lorch & Myers, 1990). A regression equation was computed for each participant with the variable Number as predictor, and dRTs as criterion. Next, a one-sample *t* test was performed on the regression weighs of all equations. Descriptive statistics are reported in Table 1.

**Table 1:** Mean and standard deviations of RTs for each condition of Experiment 2a and 2b

Numbers										
Hand	1	2	3	4	5	7	8	9	10	11
Experiment 2a										
Left	447	441	465	476	526	512	495	490	465	460
	(68.8)	(71.9)	(87.5)	(90.0)	(112)	(86.6)	(84.4)	(69.1)	(69.7)	(67.9)
Right	464	443	454	468	523	505	477	467	432	445
	(92.3)	(68.3)	(67.5)	(65.7)	(93.6)	(86.7)	(75.0)	(69.7)	(68.0)	(69.6)
Experiment 2b										
Left	528	508	546	525	525	504	535	545	544	523
	(91.8)	(72.7)	(110)	(78.8)	(75.8)	(87.9)	(75.2)	(91.9)	(92.2)	(84.8)
Right	537	500	536	506	538	510	517	524	506	514
	(95.4)	(71.3)	(88.4)	(74.2)	(90.7)	(83.2)	(77.4)	(73.8)	(78.1)	(68.7)

Values are reported in milliseconds

**Fig. 4:**
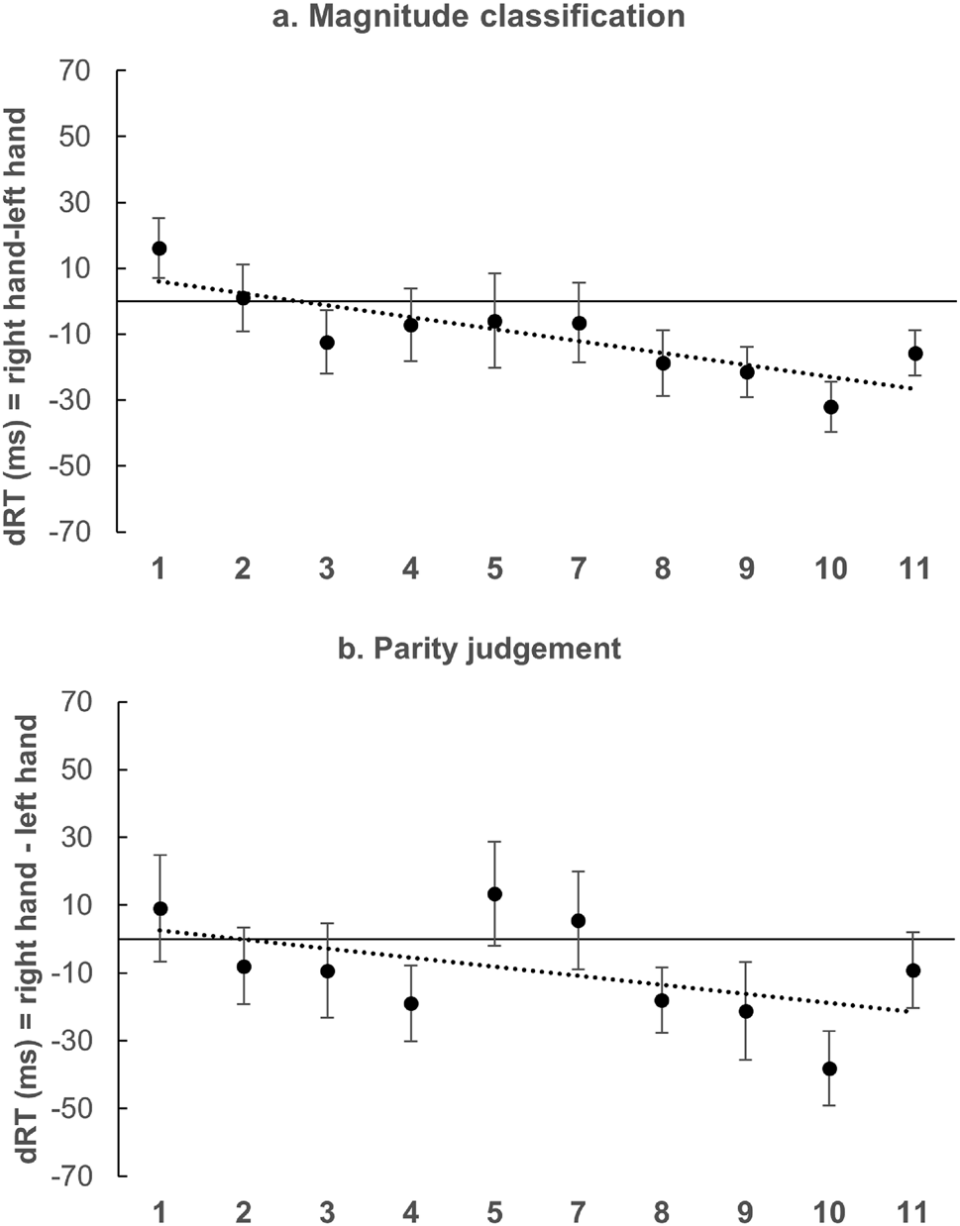
Mean dRTs (right key–left key) for every numerical stimulus in the magnitude classification (**a**) and in the parity judgement task (**b**). Positive differences indicate faster left-key responses; negative differences indicate faster right-key responses. Errors bars indicate the standard error of the mean

In Experiment 2a (magnitude classification) the 2 × 10 (Hand × Number) repeated measures ANOVA revealed a significant main effect for Hand [*F*(1, 34) = 5.67; *p* < 0.05; *η*_p_^2^ = 0.14], reflecting faster RTs with the right hand (M = 480 ms; SD = 70 ms) than with the left hand (M = 470 ms; SD = 70 ms). The analysis revealed a significant main effect for Number [*F*(9, 306) = 38.57; *p* < 0.001; *η*_p_^2^ = 0.53] with a significant quadratic trend [*F*(1, 34) = 156.67; *p* < 0.001; *η*_p_^2^ = 0.82]. A significant interaction emerged as well [*F*(9, 306) = 2.06; *p* < 0.05; *η*_p_^2^ = 0.06]. The *t* test performed on regression weighs showed that they deviated significantly from zero [M = − 3.32; SD = 9.73; *t*(34) = 2.02; *p* < 0.05; *d* = 0.34], in the direction of the SNARC effect (Fig. 4a). Finally, the occurrence of a distance effect was investigated. The average absolute RTs were calculated for the central values of the numerical range (5 and 7) and for the extreme values (1 and 11). A paired-samples t-test revealed significantly slower RTs for the central (M = 517 ms; SD = 84 ms) compared to the extreme (M = 454 ms; SD = 69 ms) numbers [*t*(34) = 12.2; *p* < 0.001; *d* = 2.06], indicating the occurrence of a distance effect.

In Experiment 2b (parity judgement), the ANOVA showed a significant main effect of Number [*F*(9, 261) = 5.32; *p* < 0.001; *η*_p_^2^ = 0.15]. There was no significant main effect of Hand [*F*(1, 29) = 3.09; *p* = 0.08; *η*_p_^2^ = 0.10] and no significant interaction [*F*(9, 261) = 1.60; *p* = 0.12; *η*_p_^2^ = 0.05]. However, the one-sample *t* test showed that the regression weights deviated significantly from zero [M = − 1.08; SD = 7.94; *t*(29) =  − 1.73; *p* < 0.05; *d* = − 0.32], in the direction of the SNARC effect (Fig. 4b).

### Discussion

Results from Experiment 2a and 2b both revealed a regular SNARC effect, showing no influence of the clockface. These results are in line with those from Experiment 1. In both cases, the context presented at the beginning of the experiment did not affect the expected results (i.e., SNB in random number generation and SNARC in magnitude classification and parity judgement). Once again, the task did not involve the retrieval of the context in working memory, and this might explain why no context-like SNAs were observed.

However, results observed in Experiment 2 are inconsistent with those studies which reported alterations of the SNARC effect due to a manipulation performed at the beginning of the experiment (Fischer et al., 2010; Mingolo et al., 2021, Experiment 2a; Shaki & Fischer, 2008). Conversely, they are consistent with the one reported by Mingolo et al., (2021, Experiment 2b), which showed that context alone cannot influence SNAs. The apparently contradictory results considered here might be attributed to the different tasks and contexts employed, as well as to the way contexts were activated. Our interpretation is that the salience of the context—when it is only elicited at the beginning of the experiment—is quite low. Consequently, the influence of the context on SNAs, if present, is modest. In order to observe an influence of the context on SNAs, we hypothesize that the context should be retrieved during task execution, and not only highlighted before the task. In a further experiment we tested this hypothesis.

## Experiment 3

Experiment 3 investigates the influence of the context on the SNARC effect when it is not only elicited before the proper experiment, but when it is further reinforced by the task. To better understand the effect of the context, in Experiment 3 task demands are manipulated in order to enhance the salience of the clockface by inducing the retrieval of the context in working memory at the moment of task execution. Experiment 3 is based on a dual task, namely participants perform both a primary and a secondary task.

Following the paradigm described in the previous experiment, the effect of the clockface context was tested through a primary task, consisting in magnitude classification for Experiment 3a and parity judgement for Experiment 3b. To enhance the salience of the context, a secondary task was added. The secondary task consisted in a Go/No-go procedure based on the spatial arrangement of the clockface, which induces participants to retrieve the context in working memory on a trial-to-trial basis to perform the task. When the context was only elicited at the beginning of the experiment (Experiment 2), a regular SNARC effect was observed. Our hypothesis is that the secondary task added in Experiment 3 will enhance the salience of the context, which will consequently influence the SNARC effect.

### Method

#### Participants

We tested 35 participants (29 women, 6 men) from the University of Trieste with a mean age of 21.11 (SD = 3.12). The sample size calculation was the same as in Experiment 2. Four participants were left-handed, and all participants had normal or corrected-to-normal vision and had always been used to exclusively read and write in a left-to-right direction. All participants reported not be affected by alcohol consumption or insufficient sleep. Written informed consent was obtained by all participants. The present experiment was conducted in accordance with the ethical standards indicated by the Declaration of Helsinki and with the approval of the University of Trieste Ethics Committee.

#### Apparatus and stimuli

**Fig. 5:**
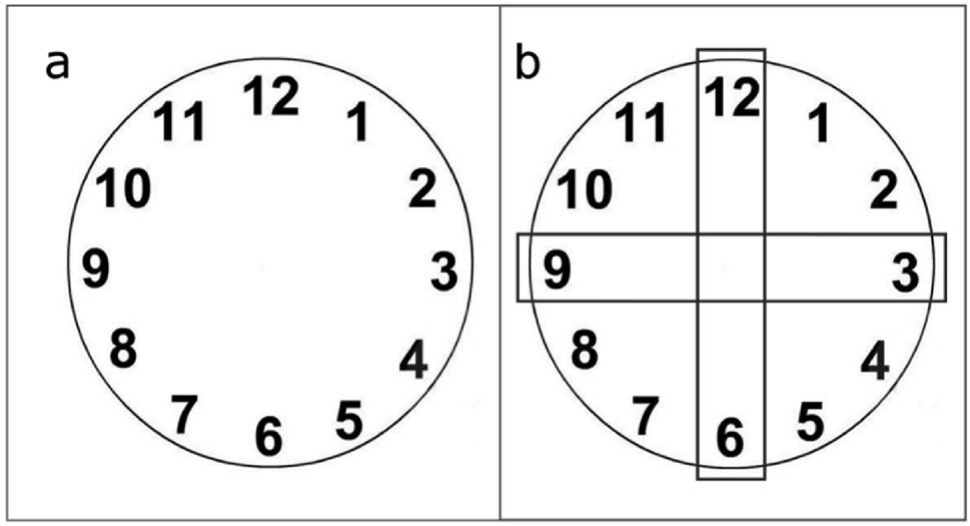
The clockface as presented to participants before the beginning of the experiment (**a**), and during practice trials (**b**) in Experiment 3

The experimental apparatus was the same as in experiments 1 and 2. The stimulus set was slightly different from that of Experiment 2; numbers 6 and 12 were this time included, for a total of 12 stimuli (1–2–3–4–5–6–7–8–9–10–11–12). See Fig. 5 for the way the context was presented.

#### Procedure

The procedure of Experiment 3 was similar to that of Experiment 2. The only difference with Experiment 2 is that a Go/No-go procedure was added to the tasks. Participants were instructed to respond to all numbers except those located on the cardinal points of the clockface (Go-stimuli were: 1–2–4–5–7–8–10–11; No-go-stimuli were: 3–6–9–12). To help participants memorize this rule, Fig. 5b was presented at each trial in the first part of the practice session, while it was not presented in the second part. Beside the Go/No-go secondary task, participants performed magnitude classification (Experiment 3a) and parity judgement (Experiment 3b). The order of presentation of the tasks was counterbalanced among participants. In the experimental session the 12 stimuli were repeated 15 times, for a total number of 180 trials for each block.

### Data analyses and results

The independent variables were Hand (left vs. right) and Number (1–2–4–5–7–8–10–11). The same analyses as in Experiment 2a and 2b were performed. In Experiment 3b (parity judgement) two participants were excluded because less than half of their RTs in at least one condition could be considered for the analyses. False alarm rate was 1.4% in Experiment 3a and 1.3% in Experiment 3b. Descriptive statistics are reported in Table 2.

**Table 2:** Mean and standard deviations of RTs for each condition of Experiment 3a and 3b

Hand	Numbers							
	1	2	4	5	7	8	10	11
Experiment 3a								
Left	541	568	606	668	690	653	634	604
	(108)	(108)	(141)	(181)	(227)	(189)	(199)	(164)
Right	572	597	613	666	662	646	596	609
	(141)	(168)	(196)	(220)	(176)	(157)	(119)	(124)
Experiment 3b								
Left	621	655	644	641	625	678	686	641
	(120)	(138)	(127)	(110)	(113)	(150)	(144)	(121)
Right	654	648	627	666	655	644	650	684
	(125)	(149)	(112)	(132)	(106)	(133)	(132)	(117)

Values are reported in milliseconds

**Fig. 6:**
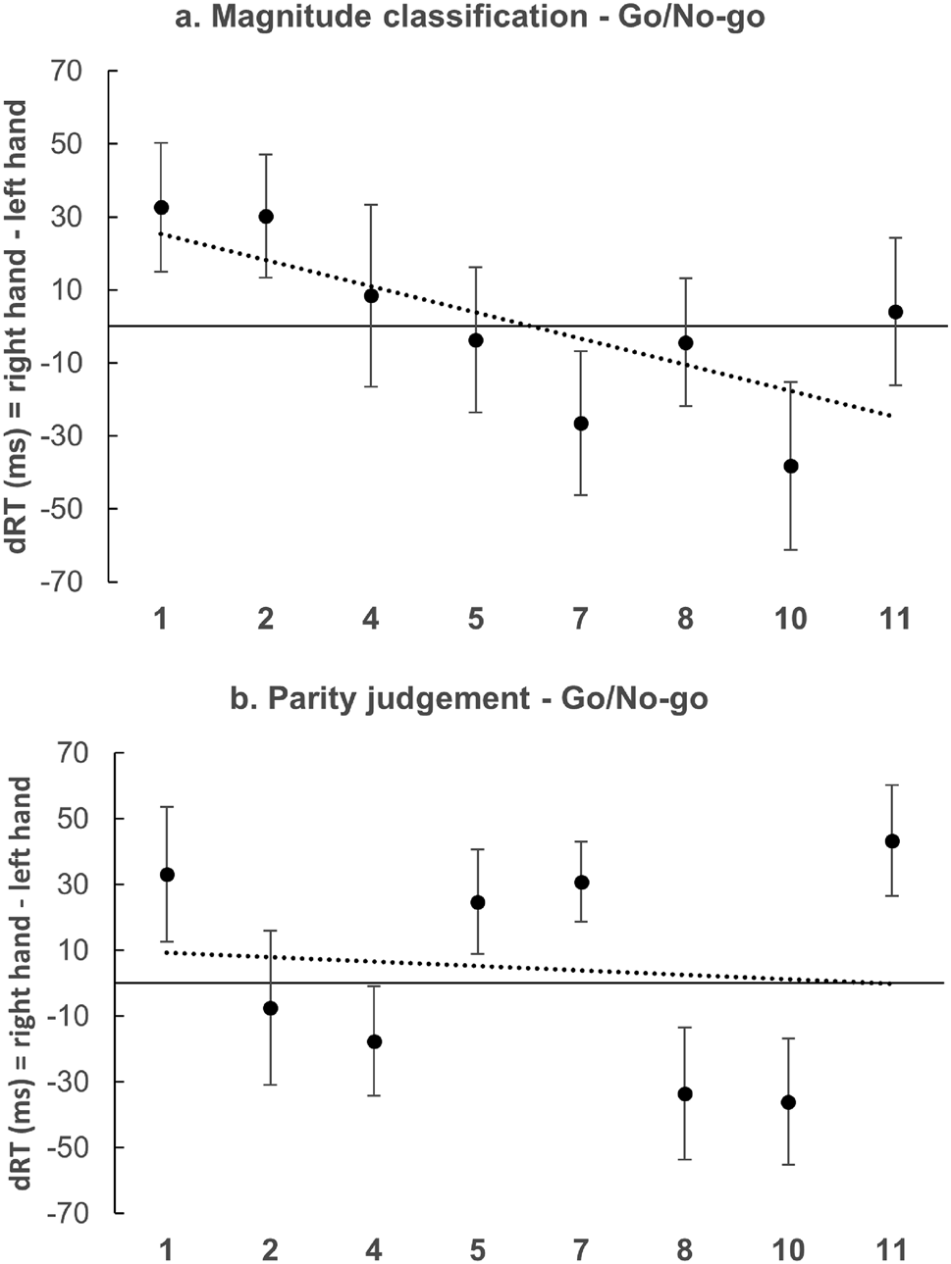
Mean dRTs (right key–left key) for every numerical stimulus in magnitude classification (**a**) and in parity judgement (**b**) in a Go/No-go procedure

In Experiment 3a (magnitude classification) the 2 × 8 (Hand × Number) repeated measures ANOVA revealed a significant main effect for Number [*F*(7, 238) = 27.66; *p* < 0.001; *η*_p_^2^ = 0.45] with a significant quadratic trend [*F*(1, 34) = 42.65; *p* < 0.001; *η*_p_^2^ = 0.56], but no significant main effect for Hand [*F*(1, 34) = 0.02; *p* = 0.88; *η*_p_^2^ = 0.001] and no significant interaction [*F*(7, 238) = 1.35; *p* = 0.23; *η*_p_^2^ = 0.04]. The one-sample *t* test conducted on individual regression weights then showed that they did not deviate significantly from zero [M = − 5.07; SD = 26.65; *t*(34) =  − 1.13; *p* = 0.27; *d* = − 0.19] (Fig. 6a). It is noteworthy that, despite the pattern of results displayed in Fig. 6a seems to be in line with the SNARC effect, in magnitude classification the regression weights do not differ significantly from zero. Finally, the occurrence of a distance effect was investigated. The average absolute RTs were calculated for the central values of the numerical range (5 and 7) and for the extreme values (1 and 11). A paired-samples t-test revealed significantly slower RTs for the central (M = 672 ms; SD = 192) compared to the extreme (M = 582 ms; SD = 121) numbers [*t*(34) = 6.45; *p* < 0.001; *d* = 1.09], indicating the occurrence of a distance effect.

In Experiment 3b (parity judgement) the ANOVA showed a significant main effect for Number [*F*(7, 224) = 2.38; *p* < 0.05; *η*_p_^2^ = 0.07], but no significant main effect for Hand [*F*(1, 32) = 0.58; *p* = 0.45; *η*_p_^2^ = 0.02]. A significant interaction occurred [*F*(7, 224) = 2.96; *p* < 0.05; *η*_p_^2^ = 0.08]. The one-sample *t* test revealed that the regression weighs did not differ significantly from zero [M = − 0.25; SD = 14.28; *t*(32) = − 0.38; *p* = 0.71; *d* = − 0.06] (Fig. 6b). The pattern of results displayed in Fig. 6b seemed to reflect the presence of a MARC effect in parity judgement. Therefore, its occurrence was investigated through a paired sample *t* test which confirmed that the mean dRTs for odd numbers differed significantly from that of even numbers [*t*(32) =  − 2.37; *p* < 0.05; *d* = − 0.41]. Moreover, all even numbers are responded faster with the right hand and all odd numbers are responded faster with the left hand.

### Discussion

Different from Experiment 2, in Experiment 3 results revealed a pattern neither in line with the SNARC effect, nor with the clockface, in both magnitude classification and parity judgement. It is noteworthy that previous studies reported SNARC-like effects using a Go/No-go procedure (e.g., Fischer & Shaki, 2016, 2017; Ginsburg & Gevers, 2015; Ginsburg et al., 2014; Lachmair et al., 2014; Pinto et al., 2019, 2021), hence we can exclude that the mere use of this procedure prevented the SNARC effect from emerging.

Our interpretation of the findings is that the secondary task induced the visuo-spatial retrieval of context in working memory. The representation elicited by the context conflicted with the primary task, and this conflict prevented the SNARC effect from emerging. We cannot completely exclude that some participants used alternative strategies, such as using the number 3 times table (i.e., 3–6–9–12, in a linear fashion) to perform the Go/No-go. However, if this was the case, there is no reason why this ordinal sequence should interfere with the SNARC effect. Furthermore, another clue supporting our interpretation comes from the MARC effect observed in Experiment 3b. This effect refers to the facilitation in responding to odd numbers with the left key and to even numbers with the right key (Nuerk et al., 2004) and is often observed in parity judgement tasks in combination with SNARC (Cipora et al., 2019). The fact that in this case only the MARC effect emerged could indicate that the context elicited through the secondary task determined a conflicting representation, which disrupted the SNARC effect. Similarly, the presence of a distance effect in Experiment 3a, which is a clear signature of a magnitude classification task, suggests that the task has been successfully completed under the Go/No-go condition. Again, we claim that the absence of a SNARC effect should be attributed to the conflicting representation of the clock face in the secondary task.

In Experiment 3 the salience of the context was enhanced only by a secondary task (i.e., Go/No-go) but not by the primary task (i.e., magnitude classification or parity judgement). In the previous literature, those experiments that showed a reversal of the SNARC effect used primary tasks in which the context was directly involved (Bächtold et al., 1998; Mingolo et al., 2021, Experiment 1). We therefore hypothesize that context is not salient enough to reverse the effect when it is only involved in a secondary task. We predict that the clockface context should be involved in the primary task to reverse the standard SNARC effect.

## Experiment 4

Experiment 4 investigated the effect of the context when it is reinforced by primary task demands. To this end, task demands in Experiment 4 rely directly on the processing of clockface information. No secondary task is used. That is, the salience of the clockface context is an intrinsic property of the task itself.

When the context was only elicited at the beginning of the experiment, a regular SNARC effect emerged (Experiments 1 and 2), while when it was reinforced by a secondary task it prevented the SNARC effect to emerge (Experiment 3). Moreover, previous experiments showed that tasks that directly rely on the context have the potential to determine SNAs consistent with the context (Bächtold et al., 1998; Mingolo et al., 2021, Experiment 1). Thus, our hypothesis is that the task used in Experiment 4 will enhance the salience of the context to the point that it reverses the SNARC effect.

### Method

#### Participants

We tested 35 participants (30 women, 5 men) from the University of Trieste with a mean age of 22.80 (SD = 6.66). The sample size calculation was the same as in Experiments 2 and 3. Two participants were left-handed, and all participants had normal or corrected-to-normal vision and were exclusively used a left-to-right reading/writing direction. All participants reported not be affected by alcohol consumption or insufficient sleep. Written informed consent was obtained by all participants. The present experiment was conducted in accordance with the ethical standards indicated by the Declaration of Helsinki and with the approval of the University of Trieste Ethics Committee.

#### Apparatus and stimuli

The same apparatus as the one in the previous experiments was employed. The numerical stimuli were the same as in Experiments 2a and 2b (i.e., 1–2–3–4–5–7–8–9-10–11).

#### Procedure

The procedure employed in Experiment 4 is the same as in Experiments 2a and 2b, except for the task demands. In Experiment 4 participants performed a “clockface-position task”, namely they had to judge whether the presented number was located on the left or the right of the central axis of the clockface. Participants responded by pressing the leftmost or the rightmost key of the response box. In the experimental session the 10 stimuli were repeated 15 times, for a total number of 150 trials for each block.

### Data analyses and results

The same analyses as in Experiments 2a and 2b were performed, with the addition of a *t* test. Five participants were excluded because less than half of their RTs in at least one condition could be considered for the analyses. Descriptive statistics are reported in Table 3.

**Table 3:** Mean and standard deviations of RTs for each condition of Experiment 4

Numbers										
Hand	1	2	3	4	5	7	8	9	10	11
Left	475	471	482	491	544	502	486	478	462	460
	(89.4)	(96.3)	(106)	(90.5)	(138)	(74.2)	(78.7)	(55.5)	(59.9)	(55)
Right	447	454	463	467	499	522	498	487	466	466
	(52.3)	(70.7)	(60.3)	(65.8)	(64.1)	(104)	(85.5)	(79.9)	(87.2)	(73.4)

Values are reported in milliseconds

**Fig. 7:**
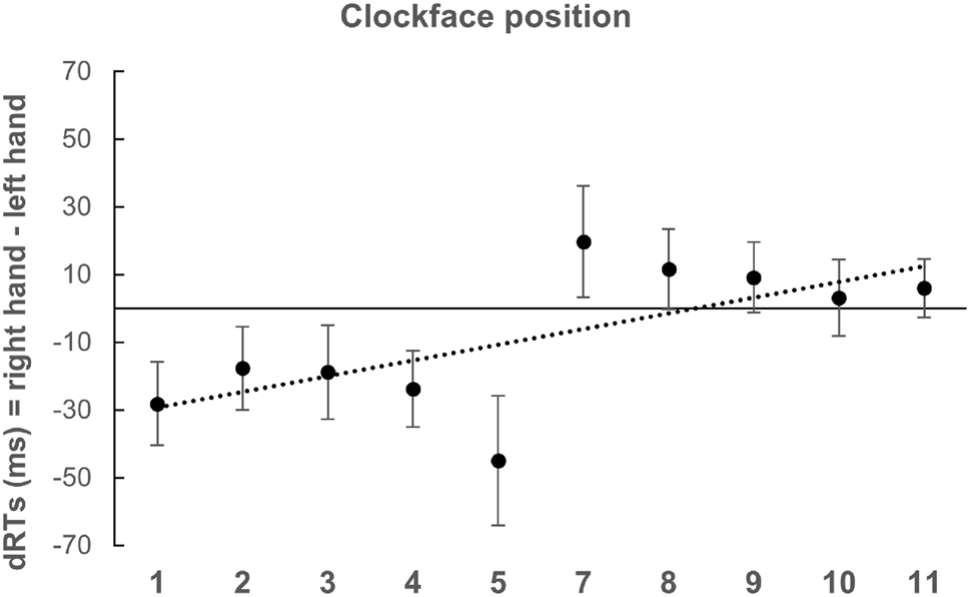
Mean dRTs (right key–left key) for every numerical stimulus in Experiment 4 (clockface position task)

The 2 × 10 (Hand × Number) repeated measures ANOVA revealed a significant main effect for Number [*F*(9, 261) = 18.96; *p* < 0.001; *η*_p_^2^ = 0.39] with a significant quadratic trend [*F*(1, 29) = 61.38; *p* < 0.001; *η*_p_^2^ = 0.68], but no significant main effect for Hand [*F*(1, 29) = 2.99; *p* = 0.09; *η*_p_^2^ = 0.09]. A significant interaction occurred [*F*(9, 261) = 2.70; *p* < 0.05; *η*_p_^2^ = 0.08]. The one-sample *t* test conducted on individual regression weights showed that they deviated significantly from zero [M = 3.01; SD = 13.06; *t*(29) = 1.78; *p* < 0.05; *d* = 0.32], in the direction of the reversed SNARC effect (Fig. 7).

Finally, the occurrence of a distance effect was investigated. The average absolute RTs were calculated for the central values of the numerical range (5 and 7) and for the extreme values (1 and 11). A paired-samples *t* test revealed significantly slower RTs for the central (M = 516 ms; SD = 83 ms) compared to the extreme (M = 462 ms; SD = 60 ms) numbers [*t*(29) = 7.53; *p* < 0.001; *d* = 1.37], indicating the occurrence of a distance effect.

### Discussion

Different from all previous experiments of this study, results from Experiment 4 indicate a response time advantage in line with the clockface, namely a reversed SNARC effect: small numbers are responded faster with the right key and large numbers are responded faster with the left key. This finding is in line with the result obtained by Bächtold et al. (1998) and with those by Mingolo et al., (2021, Exp. 1). This result indicates that, when the context is retrieved to perform the primary task, its salience is strong enough to determine the shape of a particular SNA.

Among the experiments included in the present study, Experiment 4 represents the closest replication of Bächtold et al.’s (1998) clockface condition. For this reason, we were not surprised to see that the results from the original experiment were conceptually replicated. Nonetheless, it is noteworthy that the same results emerged even though task instructions were different, suggesting that, if the context is highly salient, it influences SNAs regardless of the specific processing required by the task. Indeed, while in the original instructions the numbers were to be conceived as hours, and thus would have been processed semantically, in our experiment participants explicitly processed the spatial location of numbers. This procedure apparently determined a Simon-like effect (Simon & Rudell, 1967) in which numbers play no role. However, the occurrence of the pattern of results similar to those observed in magnitude classification tasks of Experiment 2a and 3a (i.e., a reliable distance effect suggested by the ANOVA quadratic trend and confirmed by the comparison of central vs. extreme values) indicates that number magnitude was implicitly processed. The distance effect cannot be explained by a mere Simon effect, rather it can be interpreted as the evidence of a reversed SNARC determined by a sort of mirrored MNL disposed in a clockwise direction, starting from 1 (up-right) to 11 (up-left).

## General discussion

The aim of the present study was to investigate the role of context in SNAs. In particular, the aim was to investigate how task demands can modulate the salience of the context (reflecting the level of activation of the context in working memory), and thus its effect on SNAs. To answer these questions, the same context was employed in all experiments, namely a clockface display. Conversely, task demands were manipulated across experiments to gradually enhance the level of salience of this context. Overall, the results showed that the effect of the context on SNAs is determined by its salience level, modulated here by task demands.

Experiments 1 and 2 investigated whether the context can influence the small number bias and the SNARC effect in conditions of “low salience”. To achieve this, the context was elicited at the beginning of the experiment and was not further reinforced by task demands. Results revealed a regular small number bias in Experiment 1 and a regular SNARC effect in Experiment 2. Previous studies reported alterations of the SNARC effect with contexts that elicited different reading-writing directions (Fischer et al., 2010; Shaki & Fischer, 2008) or atypical spatial-numerical configurations (Mingolo et al., 2021—Experiment 2a) at the beginning of the experiments. In contrast with these studies, the present results suggest that when a context is only elicited at the beginning of the experiment and not reinforced by task demands, it cannot influence the small number bias and the SNARC effect. This inconsistency in results might be due to the differences in the types of contexts and tasks used in the different experiments, probably because the influence of the context in these cases is weak.

To further clarify the impact of the context, Experiment 3 investigated whether the context can influence the SNARC effect in conditions of “medium salience”. To this end, the context was elicited at the beginning of the experiment and then reinforced by a secondary Go/No-go task. Results showed that the SNARC effect did not emerge. Previous studies reported the emergence of the SNARC effect despite the use of a secondary Go/No-go task (Fischer & Shaki, 2016, 2017; Ginsburg & Gevers, 2015; Ginsburg et al., 2014; Lachmair et al., 2014; Pinto et al., 2019, 2021). Therefore, this null result can be attributed to conflicting representations, one elicited by the typical representation of numbers and one elicited by the context, which was reinforced through a secondary task.

Finally, Experiment 4 investigated whether context can reverse the SNARC effect in conditions of “high salience”. To do so, the context was elicited at the beginning of the experiment and reinforced by primary task demands, without any secondary task. Results revealed a reversed SNARC effect, namely a SNA compatible with the arrangement of digits on a clockface. This result is in line with the findings by Bächtold et al. (1998), which have often been interpreted as proof that the SNARC effect is flexible and that it can be altered by contextual manipulations (Dalmaso et al., 2022; Pfister et al., 2013; Shaki & Fischer, 2008; Zhao et al., 2018). Similarly, the experiments performed on the keypad context (Mingolo et al., 2021) showed that the context can determine a consistent SNA. In that study, however, a keypad-like SNA only emerged when the configuration elicited by the context and the one elicited by the task were consistent. The present results further support this, indicating that a clockface-like SNA (like the one reported by Bächtold et al.) only emerges if task instructions reinforce the configuration elicited by the context.

The task used in Experiment 4 resembles a Simon-like task with numerical stimuli, however the results cannot be simply attributed to the Simon effect (Simon & Rudell, 1967). Indeed, the occurrence of the distance effect—similar to magnitude classification in Experiments 2a and 3a—clearly indicates that numerical magnitude was processed despite the spatial nature of the task. It is noteworthy that in the MNL the distance of central values (i.e., 5 and 7) from the centre of the configuration is smaller—by definition—than that of the extreme values (i.e., 1 and 11); while, in the clockface, the distance of the same numbers from the centre is equal. If the results merely reflected a Simon effect, no difference in absolute RTs should occur for stimuli that are equally distant in the clockface configuration. Conversely, the presence of a distance effect in Experiment 4 indicates that number magnitude was implicitly processed. The reversed SNARC pattern observed in this experiment indicates that the MNL was “mirrored” and represented in a clockface fashion, because this configuration was made salient by the primary task.

Overall, the present study suggests that an atypical context can drive SNAs only if primary task demands enhance its salience. A possible explanation for this might come from the well-known working memory account for SNAs (van Dijck & Fias, 2011). According to this account, SNAs are driven by the associations between ordinal position of numbers in working memory and space. The default association between numbers and space is consistent with the MNL (i.e., first items/left, last items/right), which explains the commonly observed SNARC effect. However, depending on task demands, temporary associations can be built to facilitate task execution.

The results from the present study are in line with Ginsburg and Gevers (2015), who showed that the ordinal position effect (namely the association between the ordinal position of items in a sequence and the response coordinates) emerges only when retrieval is required. This was observed in Experiment 3 and in Experiment 4. In both of these experiments, the retrieval of the context induced either by a secondary task (Experiment 3) or a primary task (Experiment 4) altered the SNARC effect in some way. However, the complete reversal of the effect is only observed when the retrieval of the context is induced by the primary task (Experiment 4). In this view, an atypical spatial-numerical context can change the default MNL mapping, replacing it with a more convenient context-driven mapping, only if the retrieval of the context is explicitly required by the primary task.

It is noteworthy that the task originally employed by Bächtold et al. (1998) and the one used in the present study (Experiment 4) were based on different instructions. In the original study, participants performed a semantic judgement on numbers (i.e., is the presented number a time earlier or later than 6 o’ clock?), while in our Experiment 4 participants performed a visuospatial judgement (i.e., is the presented number located on the left or on the right side of the clockface?). Therefore, these tasks rely on different working memory processes (i.e., verbal vs. visuospatial). The common factor of these experiments is that, in both cases, it was necessary to retrieve the context in working memory to solve the primary task. In this sense, finding of our Experiment 4 can be seen as an extension of the original findings, since either verbal or visuospatial instructions lead to equivalent results if they constitute a primary task. Potentially, any other primary task that requires the retrieval of the context in working memory should reveal similar results.

The results from Experiment 4 are also in line with a model that explains the role of order and magnitude in the SNARC effect (Prpic et al., 2016). This model describes an Order-Related Mechanism (ORM) responsible for the processing of stimuli’s order and a Magnitude-Related Mechanism (MRM) responsible for the processing of stimuli’s magnitude. In Experiment 4, the ordinal position of numbers elicited by the context is relevant to perform the task. Therefore, in line with the model, the ORM would be activated by both the context and the task, and this activation would induce a spatial association consistent with the clockface. For a debate on the role of order and magnitude on the genesis of the SNARC effect, see also Casasanto & Pitt (2019) and Prpic et al. (2021).

The present study contributed to investigate the mechanisms that regulate the influence of an atypical context on SNAs. The gradual manipulations of task demands helped understand which aspects of the task enhance the salience of the context, contributing to its influence on SNAs. However, the design of the study did not allow to directly compare the results throughout the experiments. Future studies should employ a paradigm that manipulates salience within the same primary task. Moreover, the secondary task used to enhance the salience of the context at a medium level could have led the participants to use alternative strategies to perform the primary task. In this regard, further studies investigating the effect of context on SNAs should employ tasks which can be controlled for the possible strategies used by participants.

Overall, it was clarified that an atypical context does not influence SNAs if not further reinforced by the task. The present study extends the knowledge on SNA by unveiling the mechanisms behind the original clockface finding (Bächtold et al., 1998), which would be responsible for the flexibility of these effects. Here we clarify that the original clockface finding would not have been observed if the task did not involve the clockface configuration to be performed. Namely, the retrieval of the context in working memory—induced during the primary task—would be the crucial mechanism underlying the original effect reported by Bächtold et al. From a methodological perspective, the present study aimed to raise attention over possible biases that could occur when interpreting results in SNAs research. Namely, when investigating the effect of an atypical context over SNAs, particular attention should be paid to task characteristics and, where appropriate, the effect of the context should be investigated in combination with different tasks.

## Conclusions

The previous literature showed how SNAs in general and the SNARC effect in particular, are flexible and can be modulated by the context in which stimuli are presented. Bächtold et al. (1998) reported the reversal of the SNARC effect determined by the influence of an atypical context, namely a clockface. However, the mechanisms that regulate this modulation were not clear. In the present study, we investigated whether and how the salience of an atypical spatial-numerical context can alter SNAs. To this aim, the clockface was presented as context and its salience level was gradually increased by task demands across four experiments. Results highlighted that when the task does not enhance the salience of the clockface context, a regular SNARC effect emerges, which indicates that the context does not influence it. Secondly, when the task enhances the salience of the context at a medium level, conflict between different representations seem to prevent the SNARC effect from emerging. Finally, when the task enhances the salience of the context at the highest level, namely when it is based on the same configuration as the context, a reversal of the SNARC effect emerges in consistency with the context. In a nutshell, the results of the present study highlight that context can shape SNAs only when primary task demands make it sufficiently salient and, thus, active in working memory.

## Data Availability

Raw data and study materials are available at the following link: https://osf.io/y2uce/?view_only=801a434d274d47f89ad5c768656aaeeb.
